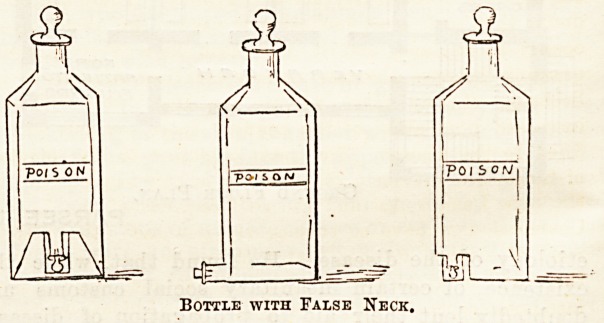# Poison Bottles

**Published:** 1895-08-24

**Authors:** 


### PRACTICAL DEPARTMENTS.

### POISON BOTTLES.

Whenever some lamentable accident occurs through carelessness, or by one of those curious mistakes through which every now and then a life is sacrificed, and carbolic acid or such like taken or administered "in mistake," suggestions invariably flow from all sides whereby repetitions may be avoided. It is certainly sufficiently demonstrated over and over again that adequate care is not taken, in private households at any rate, in keeping dangerous poisons in receptacles which cannot by any means be confused with those containing harmless preparations.

With the ordinary blue, ribbed poison bottle we are all

Ordinary Poison Bottle.

well acquainted, but it is rightly thought that the difference thus made is not sufficiently distinctive, inasmuch as other more innocent bottles are also made of ribbed glass, and therefore in a dim light little protection is afforded. Many proposals have been made and expedients suggested to attain the desired end, and the accompanying illustrations represent some of those which have been especially brought to our notice. If any of our readers seeing these find that other ideas suggest themselves, we shall be glad to give description and drawings in these columns, in the hope that ultimately some definite distinction may be universally adopted, and so bring about a reform in poison dispensing which is sadly needed.

Inquiries at the Aire and Calder Bottle Company, the well-known glass bottle manufacturers, Upper Thames-street, prove the fact that whatever brilliant inventions may have resulted, none of these have been so far largely adopted. The ordinary "blue" bottle, in various sizes, is the only kind of poison speciality known to the firm. By their kind permission, we give a sketch of the bottle in question. (No. 1.)

Sketch No. 2 represents a novel idea, brought out by Mr. G. Padmore, 84, Regent-street. We have received one of these bottles as a specimen, and sketched it as here shown. We are bound to confess that although the apparatus would undoubtedly be successful in recalling the attention of any-one using the bottle to the nature of its contents (which can only be properly poured out by the exercise of some care in keeping the "shield" out of the way), yet it is a somewhat clumsy contrivance in itself, and one which we fear is not likely for that reason to find very extensive favour with the general public. Our illustration shows the protecting cap in two positions.

Bottle No. 3 is given in three slightly different variations. The idea, which comes from Dr. Stocker, of Forest Gate, is that the apparent neck and stopper of the bottle shall be merely a dummy, the real orifice being at the bottom of the bottle. In this way a complete reversing of the whole bottle is necessary before the liquid can be poured out. Our sketches

Bottle with Protective Shield.

are taken from a rough outline on paper, and they are, therefore, capable of improvement. Dr. Stocker proposes that the true stopper shall be of glass and indiarubber combined, so arranged that only the glass portion is in actual contact with the fluid.

Other suggestions have also reached us from time to time, one of the best being that a special shape of bottle should be entirely restricted to poison use, such as a triangular one. It has been proposed, too, that the glass of which the bottles are made shall be of such a rough exterior as to infallibly remind those touching them, whether in the dark or daylight, of their special purpose.

It certainly ought to be possible by some general and easily recognised distinction to prevent such accidents as that which occurred not so long ago in the case of Professor Tyndall.

Bottle with False Neck.

Some mistakes, such as that which has recently been commented upon in the papers, where the wholesale dealers from whom a chemist procured his drugs had mixed together phenacitin and strychnine in some inexplicable way, with two fatal results, cannot be guarded against on the part of the general public, but there can be no doubt that very great and culpable carelessness exists in many households in connection with poison, and the reckless way in which laudanum, chloroform, carbolic acid, and other dangerous drugs are left with harmless medicines, often not even in the dark blue bottles wherein they are supplied in the first instance, leads those who at all realise what terrible results may consequently happen, only to marvel that such fatalities are not more frequently heard of.

## Figures and Tables

**Figure f1:**
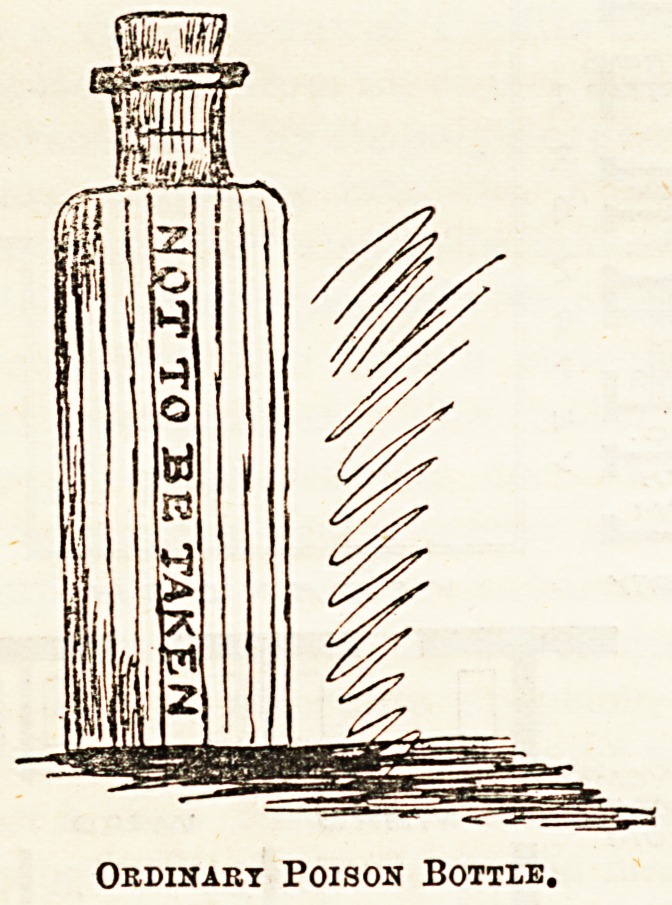


**Figure f2:**
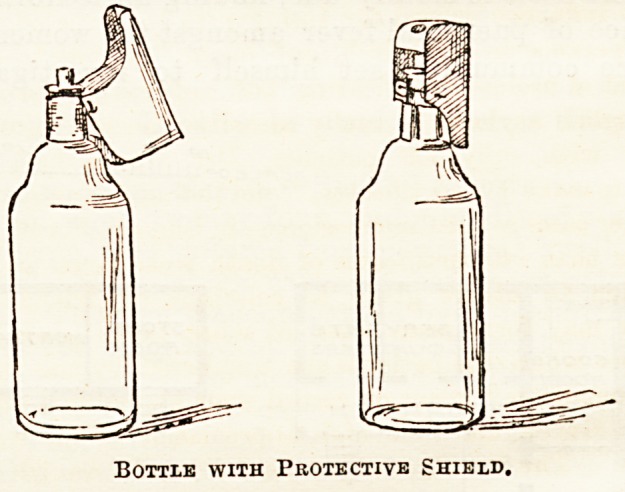


**Figure f3:**